# Gap Junction Dependent Cell Communication Is Modulated During Transdifferentiation of Mesenchymal Stem/Stromal Cells Towards Neuron-Like Cells

**DOI:** 10.3389/fcell.2020.00869

**Published:** 2020-08-31

**Authors:** Nadine Dilger, Anna-Lena Neehus, Klaudia Grieger, Andrea Hoffmann, Max Menssen, Anaclet Ngezahayo

**Affiliations:** ^1^Department of Cell Physiology and Biophysics, Institute of Cell Biology and Biophysics, Leibniz University Hannover, Hanover, Germany; ^2^Institute of Experimental Hematology, REBIRTH Research Center for Translational and Regenerative Medicine, Hannover Medical School (MHH), Hanover, Germany; ^3^Graded Implants and Regenerative Strategies, Department of Orthopedic Surgery, Hannover Medical School, Hanover, Germany; ^4^Lower Saxony Centre for Biomedical Engineering, Implant Research and Development (NIFE), Hanover, Germany; ^5^Department of Biostatistics, Institute of Cell Biology and Biophysics, Leibniz University Hannover, Hanover, Germany; ^6^Center for Systems Neuroscience, University of Veterinary Medicine Hannover, Hanover, Germany

**Keywords:** mesenchymal stem/stromal cells, transdifferentiation, neuron, small molecules, gap junctions, connexins, human

## Abstract

*In vitro* transdifferentiation of patient-derived mesenchymal stem/stromal cells (MSCs) into neurons is of special interest for treatment of neurodegenerative diseases. Although there are encouraging studies, little is known about physiological modulations during this transdifferentiation process. Here, we focus on the analysis of gap junction dependent cell-cell communication and the expression pattern of gap junction-building connexins during small molecule-induced neuronal transdifferentiation of human bone marrow-derived MSCs. During this process, the MSC markers CD73, CD90, CD105, and CD166 were downregulated while the neuronal marker Tuj1 was upregulated. Moreover, the differentiation protocol used in the present study changed the cellular morphology and physiology. The MSCs evolved from a fibroblastoid morphology towards a neuronal shape with round cell bodies and neurite-like processes. Moreover, depolarization evoked action potentials in the transdifferentiated cells. MSCs expressed mRNAs encoding Cx43 and Cx45 as well as trace levels of Cx26, Cx37- and Cx40 and allowed transfer of microinjected Lucifer yellow. The differentiation protocol increased levels of Cx26 (mRNA and protein) and decreased Cx43 (mRNA and protein) while reducing the dye transfer. Cx36 mRNA was nearly undetectable in all cells regardless of treatment. Treatment of the cells with the gap junction coupling inhibitor carbenoxolone (CBX) only modestly altered connexin mRNA levels and had little effect on neuronal differentiation. Our study indicates that the small molecule-based differentiation protocol generates immature neuron-like cells from MSCs. This might be potentially interesting for elucidating physiological modifications and mechanisms in MSCs during the initial steps of differentiation towards a neuronal lineage.

## Introduction

Mesenchymal stem/stromal cells (MSCs) are multipotent adult stem cells with the ability to self-renew and to differentiate into osteoblasts, chondrocytes and adipocytes *in vivo* (Berry et al., 1992; Herbertson and Aubin, 1997; Kuznetsov et al., 1997). They can be cultivated for multiple passages. Besides their natural differentiation potential, they can artificially be transdifferentiated into cells of other lineages like cardiomyocytes (Kawada et al., 2004; Huang et al., 2015; Shi et al., 2016) or neuronal cells (Ma et al., 2011; Feng et al., 2014; Qin et al., 2015; Hwang et al., 2017). Therefore, MSCs are thought to open new perspectives for regenerative medicine, as it may be possible to regenerate diverse cell types of the human body from patient-derived MSCs (Phinney and Prockop, 2007; Mollinari et al., 2018).

Neurons are post-mitotic cells that cannot be donated by healthy persons. Therefore, transdifferentiation of neurons from patient-derived cells could be an option in treatment of neurodegenerative diseases. Concerning clinical applications, the usage of small molecules offers perspectives of converting without genetically modifying cells and therefore lower the patients’ risk (Qin et al., 2017). Regarding basic research, transdifferentiation offers possibilities to gain more insights into physiological modifications during cell differentiation.

Gap junction mediated cell-cell communication is known to be modulated during neuronal differentiation. Gap junctions are intercellular channels which can assemble to gap junction plaques. They directly connect the cytoplasm of adjacent cells, thus permitting a bidirectional exchange of molecules up to 1–2 kDa like ions, metabolites or second messengers (Söhl and Willecke, 2004; Goodenough and Paul, 2009). Gap junction mediated cell-cell communication thereby allows the progression of electrical and chemical signals in a tissue and has an important impact on physiology, growth and differentiation of cells (Söhl et al., 2005). Gap junctions are composed of oligomerized integral membrane proteins called connexins (Cx), of which 21 isoforms have been identified in humans. The connexin expression pattern is tissue specific and is regulated during cell differentiation (Nielsen et al., 2012).

MSCs are extensively gap junction-coupled and mainly express Cx43, as well as Cx40 and Cx45 (Dorshkind et al., 1993; Bodi et al., 2004; Valiunas et al., 2004). Neurons are also coupled by gap junctions (Lo Turco and Kriegstein, 1991; Bittman et al., 1997) which are mainly composed of the connexins Cx26, Cx30.2, Cx45 and particularly Cx36 (Leung et al., 2002; Kreuzberg et al., 2008; Eugenin et al., 2012; Su et al., 2017). Amongst these, Cx36 is the most prominent neuronal connexin in adult electrical synapses and plays important roles in the developing brain (Belluardo et al., 2000; Condorelli et al., 2000). Gap junction mediated cell-cell communication seems to be essential for neurogenesis, during which the expressed connexin isoforms change (Bosone et al., 2016; Swayne and Bennett, 2016). Along their differentiation, neural progenitor cells need to down-regulate multiple connexin isoforms, especially that of Cx43 and become less gap junction-coupled (Rozental et al., 2000; Rinaldi et al., 2014).

In this report we used small molecule-based transdifferentiation protocols defined by Bi et al. (2010) and Aguilera-Castrejon et al. (2017) to induce the conversion of human bone marrow-derived MSCs into neuronal cells. Analyzing the gap junction coupling during the transdifferentiation process validated that the induced cells are suitable to study physiological modulations in MSCs overcoming mesenchymal cell fate.

## Materials and Methods

### Cell Culture

Human bone marrow-derived MSCs from a healthy female donor were isolated and cultured as described by Jungwirth et al. (2018).

### Neuronal Induction of MSCs

MSCs were grown in cell culture plates or seeded onto collagen I-coated glass coverslips and cultivated until they reached a confluency of 70%. Differentiation was induced with differentiation media developed by Bi et al. (2010) and Aguilera-Castrejon et al. (2017) (Supplementary Table 1).

The protocol of Bi et al. (2010), further referred to as NIM-1 protocol, describes a pre-induction of MSCs in MSC medium with 1 μM retinoic acid (RA) for 24 h after which the neural differentiation was induced with a neural induction medium (NIM-1) for additional 24 h.

Following the protocol of Aguilera-Castrejon et al. (2017), MSCs were cultivated in a neural induction medium (NIM-2) for 7 days (referred to as 7d NIM-2 protocol). Additionally, the incubation time in NIM-2 was prolonged to 30 days (named 30 d NIM-2 protocol). Before further electrophysiological experiments, cells derived from the 30 d NIM-2 protocol were transferred into a maturation medium (MAT; Hu et al., 2015) for 24 h (referred to as 30 d NIM-2 1d protocol).

Protein and RNA isolation, immunocytochemical staining and physiological analysis were performed after each protocol.

### Quantitative Real-Time PCR

Quantitative real-time PCR (qRT-PCR) was used to quantify mRNA expression of MSC and neuronal markers as well as different connexin isoforms. Undifferentiated MSCs were used as control. RNA was isolated using the PeqGOLD Total RNA kit (Peqlab). Thereafter, RNA was reverse transcribed into cDNA using the Maxima First Strand cDNA synthesis kit (Thermo Fisher Scientific, Waltham, MA, United States). 12.5 ng of cDNA were used as template for the qRT-PCR which was performed with the KAPA SYBR FAST Universal mastermix (Kapa Biosystems) in a volume of 10 μL. All primer data are given in Supplementary Table 3. Correct amplification was confirmed by sequencing. For each sample and primer pair three technical replicates were analyzed. Each qRT-PCR was run at least three times with cDNA of independent differentiations. The mRNA level of the gene of interest relative to the housekeeping gene *RPS29* was calculated by 2^–ΔCt^ method.

### Immunocytochemistry

The cells were washed with PBS, fixed with 4% formaldehyde or ice-cold acetone/methanol (1:1) and permeabilized and blocked simultaneously in 0.1% Triton X-100 and 1% BSA in PBS. The cells were incubated overnight at 4°C in primary antibody solutions of anti-Cx26 (1:1,000, MABT198, Merck Millipore), anti-Cx43 (1:4,000, C6219, Sigma-Aldrich), anti-Cx45 (1:50, 5C7G1, Thermo Fisher Scientific), anti-Tuj1 (1:1000, 2G10, ab78078, Abcam), anti-Nestin (1:200, SP103, ab105389, Abcam), anti-Map2 (1:50, 4542, Cell Signaling Technology) and anti-NeuN (1:100, A60, MAB377, Merck Millipore). The secondary iFluor^TM^488-conjugated anti-mouse (1:500, 16448, AAT Bioquest) and anti-rabbit antibodies (1:500, 16608, AAT Bioquest) together with 2 μM DAPI (Sigma-Aldrich) were added to the cells for 1 h at 37°C. The cells were imaged with a Nikon Eclipse TE2000-E C1 confocal laser-scanning microscope (Nikon GmbH).

### Western Blot

Total protein of the cells was isolated and the protein concentration was estimated using a Bradford assay (Sigma-Aldrich). 20 μg of total protein were separated by SDS-PAGE and transferred onto a nitrocellulose membrane using semi-dry transfer. Therafter, the membrane was blocked in 5% non-fat dry milk powder in PBS containing 0.1% Tween 20 (PBS-T). Primary antibody incubation was performed overnight at 4°C. The primary antibodies anti-β-tubulin (1:2,000, T4026, Sigma-Aldrich), anti-GAPDH (1:100,000, ab181602, Abcam), anti-Cx43 (1:7,500, C6219, Sigma-Aldrich), anti-Cx26 (1:1,000, MABT198, Merck Millipore), anti-Cx45 (1:500, 5C7G1, Thermo Fisher Scientific), anti-Tuj1 (1:2,000, ab78078, Abcam) and anti-NeuN (1:200, MAB377, Merck Millipore) were diluted in PBS-T. Depending on the molecular weight of the target proteins, either GAPDH or β-tubulin was used as internal standard. The following day, the secondary anti-rabbit antibody (A9169, Sigma-Aldrich), diluted in PBS-T (1:40,000), was added to the membrane for 1 h at room temperature, followed by the secondary anti-mouse antibody (A9044, Sigma-Aldrich), diluted 1:40,000 in PBS-T for another 1 h incubation at room temperature. The membrane was imaged with ECL substrate. The western blot experiments were performed four times and analyzed with the gel analyzer tool of Fiji (Schindelin et al., 2012). The measured band intensities were first normalized to the loading control (GAPDH or β-tubulin) and secondly to the undifferentiated MSC samples.

### Dye Transfer Experiments

Gap junction coupling was analyzed by dye transfer experiments with Lucifer Yellow (LY) lithium salt (Biotium). Cells grown on coverslips were transferred into a perfusion chamber filled with 500 μL of a bath solution (140 mM NaCl, 5 mM KCl, 10 mM HEPES, 1 mM MgCl_2_, 10 mM glucose, 2 mM CaCl_2_ at pH 7.4 and 295 mosmol/L). A whole-cell patch-clamp configuration was established onto the central cell of the observed area using an EPC 10 USB double patch-clamp amplifier (HEKA). The patch pipette was filled with a LY (1 mg/mL)-containing pipette solution (145 mM K-gluconate, 5 mM KCl, 10 mM HEPES, 2.5 mM MgATP, 5 mM glucose, 0.5 mM Na_2_ATP, 1 mM EGTA, 0.5 mM CaCl_2_ at pH 7.4 and 295 mosmol/L). The dye was allowed to diffuse into the patched cell for 10 min before the pipette was removed. LY was excited at 410 nm. For each experiment, fluorescence images were captured before establishing the whole-cell patch-clamp configuration, during the experiment and after removal of the pipette. The micrographs were analyzed using Fiji (Schindelin et al., 2012). Images taken before the experiment were subtracted from images captured after pipette removal. To quantify cell coupling, the integrated density of the fluorescence signal was measured in all cells except for the patched cell and then normalized to the integrated density of the patched cell. This ratio will further be referred to as LY dye coupling rate.

### Electrophysiology

The whole-cell configuration was established onto a cell using the same setup and conditions as described above for dye transfer experiments. The bath and pipette solutions were identical except for LY lacking in the pipette solution. Patch pipettes showed electrical resistances of 4–6 MΩ. Cells were clamped to −60 mV and action potentials were induced in current-clamp mode by injecting depolarizing currents.

### Statistical Analysis

All statistical analyses were run using R and linear models were fit by the lm() function (R Core Team, 2019). Model based least-square means were compared using the emmeans package version 1.3.4 (Lenth, 2019) with *α* = 0.05. All data sets and corresponding R-code are available under https://github.com/MaxMenssen/Dilger_et_al_2020.

Analyzing the qRT-PCR data, 2^–ΔCt^ values were ln-transformed and modeled based on runs and treatments separately for each gene of interest. Least-square means were compared between the control group and further treatments.

2^–ΔCt^ values for the CBX data were split by genes of interest. For each gene, ln-transformed 2^–ΔCt^ values were modeled based on run, treatments, CBX concentrations and the interaction between treatments and concentrations. Comparisons of means were run such that means were compared against MSC without CBX and against 7 d NIM-2 without CBX.

Western blot data was split by proteins. Normalized protein expression was ln-transformed and modeled depending on runs and treatments. Since normalization set control values to one, the treatment means were tested to be significantly different from one.

For the dye transfer data, the minimum coupling rate was added to all coupling rates and was ln-transformed and modeled based on different treatments. Subsequently, mean comparisons between control and treatments were run.

## Results

**FIGURE 1:**
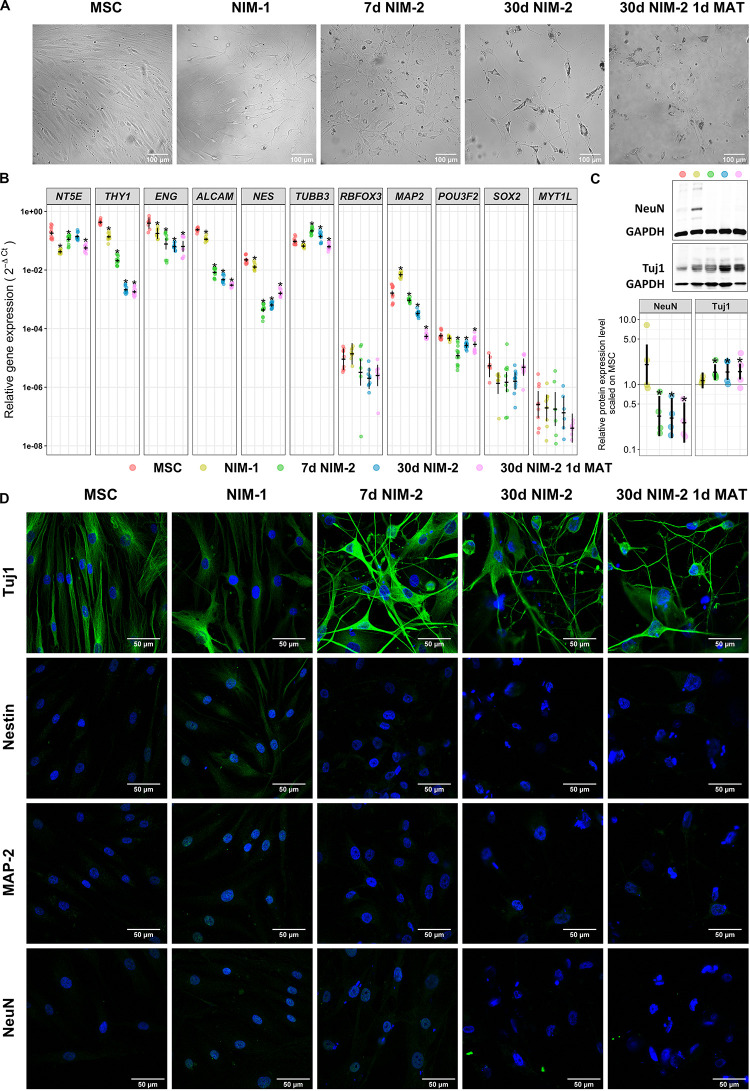
Transdifferentiation of MSCs into neuron-like cells. **(A)** Bright-field images of MSCs before and after the differentiation procedures. Note the morphology changes of the cells into cells with round cell bodies and long cell processes. The scale bars represent 100 μm. **(B)** Gene expression analysis of MSC markers (*NT5E*, *THY1*, *ENG*, *ALCAM*) and neuronal markers (*NES*, *TUBB3*, *RBFOX3*, *MAP2*, *POU3F2*, *SOX2*, *MYT1L*) by qRT-PCR. **(C)** Western blotting of the neuronal markers Tuj1 and NeuN. Exemplary blots are shown in the upper panel. A quantitative analysis normalized to GAPDH and control MSCs is shown in the lower panel. **(D)** Immunocytochemistry staining of the neuronal markers Tuj1, Nestin, MAP2 and NeuN in MSCs and the differentiated cells. The protein of interest is visualized by secondary antibodies labeled with iFluor^TM^488 (green) while the nuclei are stained with DAPI (blue). The scale bars represent 50 μm in panels **(B,C)** (lower panel) the original data points are plotted in colorful dots. The horizontal lines indicate model-based least-square means together with their 95%-confidence intervals (vertical lines). The * marks significant mean differences (α = 0.05) between MSCs and the differentiated cells.

The goal of this study was to analyze the connexin expression and gap junction mediated cell-cell communication during transdifferentiation of MSCs into neuronal cells. MSCs from human bone marrow proliferated and formed an adherent monolayer of cells with fibroblast-like morphology (Figure 1A). We used the protocols NIM-1, 7 d NIM-2, 30 d NIM-2 and 30 d NIM-2 1 d MAT, based on the publications of Bi et al. (2010) and Aguilera-Castrejon et al. (2017), to develop MSCs into neuronal cells.

Following the conversion of the MSCs into neuron-like cells, we analyzed changes in cell morphology and expression level of the MSC markers CD73 (*NT5E*), CD90 (*THY1*), CD105 (*ENG*) and CD166 (*ALCAM*), the neuronal stem cell marker Nestin (*NES*), the neuronal markers Tuj1 (*TUBB3*), NeuN (*RBFOX3*) and MAP-2 (*MAP2*) and the transcription factors Brn-2 (*POU3F2*), SOX-2 (*SOX2*) and MyT1-L (*MYT1L*) (Figure 1, for gene/protein equivalents also see Supplementary Table 2).

The applied transdifferentiation protocols reduced proliferation and induced a morphological development from fibroblast-like cells to cells with round cell bodies and long cell processes (Figures 1A,D). At mRNA level, the MSCs expressed *NT5E*, *THY1*, *ENG* and *ALCAM* as well as *NES* and *TUBB3.* The NIM-1 protocol down-regulated *NT5E*, *THY1*, and *ALCAM*, while *MAP2* was upregulated. Differentiating the MSCs with the NIM-2 protocols reduced the mRNA levels of *NT5E*, *THY1*, *ENG*, *ALCAM*, *NES*, *MAP2* and *POU3F2*. The induction with the 30 d NIM-2 and 30 d NIM-2 1 d MAT protocols reinforced the down-regulation of *THY1* and *ALCAM* (Figure 1B). The muscle specific markers *ACTA2*, *TAGLN*, *MYL2* and *SMYD1* were not upregulated at mRNA level during differentiation.

At protein level, the expression of Nestin, MAP-2, Tuj1 and NeuN was analyzed by Western blotting and immunocytochemical staining (Figures 1C,D). Immunocytochemistry revealed no expression of Nestin, MAP-2 and NeuN in MSCs which was not altered by the NIM-2 differentiation protocols. Only the NIM-1 protocol resulted in slightly increased expression of NeuN, which was mainly located in the nucleus, and of Nestin. Tuj1 was detectable in MSCs, while the differentiation protocols 7 d NIM-2, 30 d NIM-2 as well as 30 d NIM-2 1 d MAT increased the Tuj1 signal after differentiation (Figure 1D). To quantify the protein expression, Western blotting experiments were performed of the most unambiguously expressed neuronal marker Tuj1, which is a marker for immature neurons. For comparison, NeuN, a marker for mature neurons, was also analyzed by Western blotting experiments. The expression of Tuj1 in MSCs and its upregulation by the differentiation procedures were confirmed (Figure 1C). NeuN was barely detectable in MSCs via Western blot but a basal expression was measurable. This was not the case when the cells were differentiated by NIM-2 which down-regulated NeuN. Only the NIM-1 protocol had an inducing effect on NeuN although the samples were heterogeneous in their expression level (Figure 1C).

**FIGURE 2:**
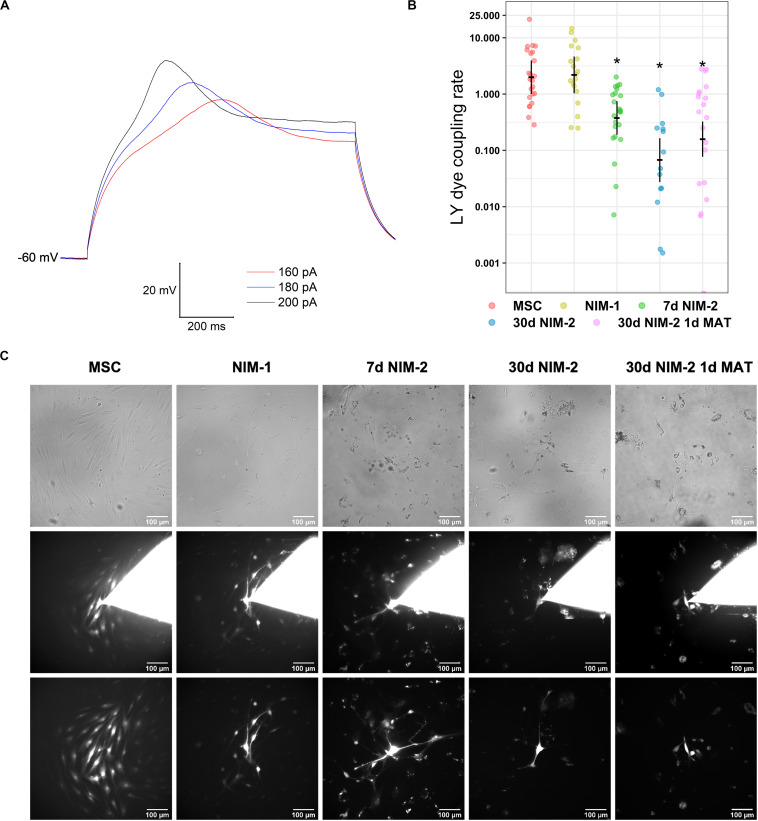
Electrophysiological properties and gap junction coupling in differentiated MSCs. **(A)** Membrane potential recordings in a 30 d NIM-2 1 d MAT-differentiated cell measured in the whole-cell patch-clamp configuration. Action potentials were evoked by the injection of depolarizing currents of 160–200 pA. **(B)** Quantification of the dye transfer experiments. The original data points are plotted in colorful dots. The horizontal lines indicate model-based least-square means together with their 95%-confidence intervals (vertical lines). The * marks significant mean differences (α = 0.05) between MSCs and the differentiated cells. **(C)** Exemplary micrographs of LY dye transfer experiments. Phase contrast images captured before the experiment are shown in the first row. The second row shows the cells during the dye transfer experiment with the patch pipette still attached onto the cell. In the third row, micrographs of the dye transfer experiment are displayed, which were captured after removing the patch pipette to evaluate the degree of gap junction coupling. The scale bars indicate 100 μm.

To further characterize the differentiation of the MSCs into neuron-like cells, electrophysiological measurements were performed. MSCs differentiated by the 30 d NIM-2 1 d MAT protocol showed resting membrane potentials of −60 mV and responded to the injection of depolarizing currents by firing action potentials. The action potentials were scarcely overshooting and the cells did not sufficiently repolarize to allow repetitive action potentials during the depolarization interval (Figure 2A).

Gap junctions are known to have an impact on cell differentiation and are essential for electrical synapses in neuronal networks. We therefore analyzed the influence of the gap junction dependent cell-to-cell communication during the neuronal transdifferentiation of MSCs into neuron-like cells.

MSCs were coupled by gap junctions as LY was able to diffuse into numerous neighboring cells during dye transfer experiments. The differentiation protocols 7 d NIM-2, 30 d NIM-2 and 30 d NIM-2 1 d MAT reduced the capability of dye transfer, whereas it was not affected by the NIM-1 protocol (Figures 2B,C). Although time-dependent effects cannot be completely excluded, the results suggest an effect on gap junction coupling.

**FIGURE 3:**
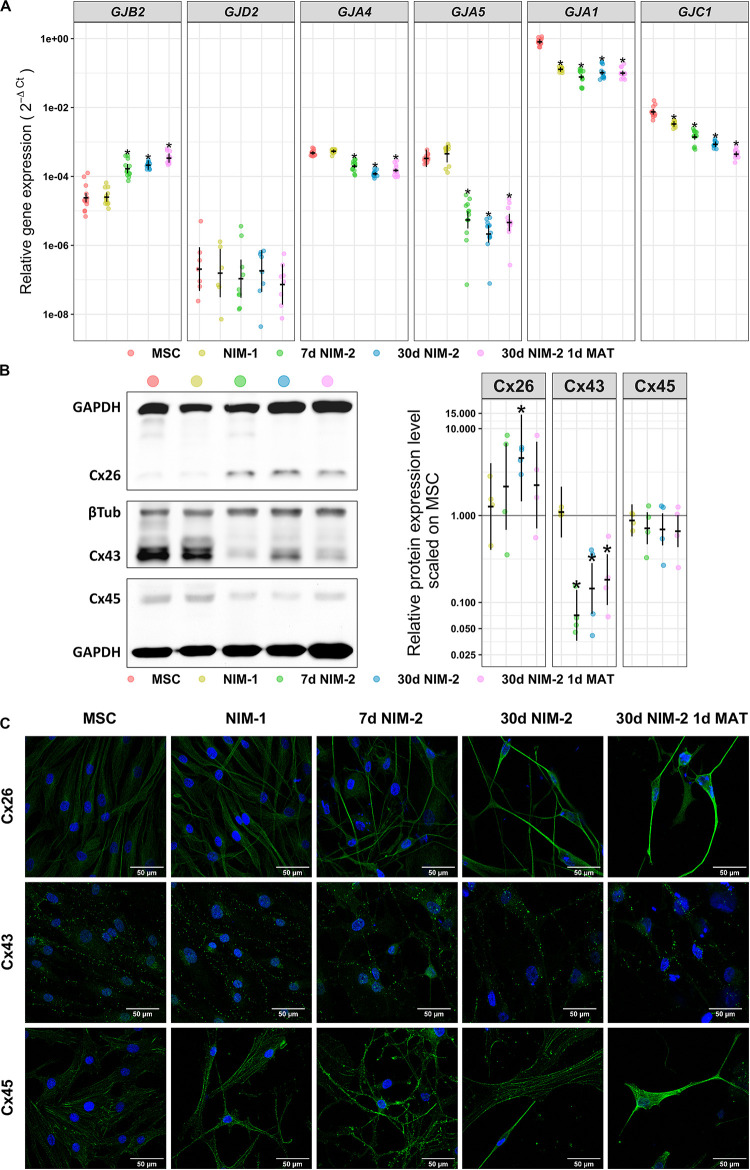
Connexin expression in MSCs and NIM-1- and NIM-2-differentiated cells. **(A)** qRT-PCR analysis of *GJB2, GJA4*, *GJA5*, *GJA1*, and *GJC1* as well as neuronal *GJD2*. **(B)** Western blot analysis of the connexin expression in MSCs and differentiated cells. Exemplary blots are shown on the left and its quantification on the right. **(C)** Immunocytochemistry staining of Cx26, Cx43 and Cx45 (green) and the nuclei (DAPI, blue). The scale bars represent 50 μm. In panels **(A,B)** (right panel) the original data points are plotted in colorful dots. The horizontal lines indicate model-based least-square means together with their 95%-confidence intervals (vertical lines). The * marks significant mean differences (α = 0.05) between MSCs and the differentiated cells.

In order to identify the expressed connexins forming the gap junctions, qRT-PCR and Western blotting experiments were performed as well as immunocytochemistry staining of the most prominent connexins (Figure 3). In MSCs the qRT-PCR results revealed strong expression of *GJA1* (Cx43), while *GJC1* (Cx45) was less present. Even weaker expressed were *GJA4* (Cx37), *GJA5* (Cx40) and *GJB2* (Cx26). *GJD2* (Cx36) was barely detectable. The NIM-1 protocol down-regulated *GJA1* and *GJC1* but did not affect the other connexin isoforms. The differentiation with the NIM-2 protocols down-regulated *GJA4*, *GJA5*, *GJA1* and *GJC1* whereas the *GJB2* expression was up-regulated. The mRNA level of *GJD2* was not affected by any of the applied protocols (Figure 3A).

The results of the qRT-PCR analysis of *GJB2*, *GJA1* and *GJC1* were re-examined at protein level. In contrast to the qRT-PCR results, Cx45 was barely detectable in MSCs by Western blot and its expression did not change after treatment with the NIM-1 or NIM-2 protocols. On the other hand, Cx43 was clearly detectable and appeared to be strongly expressed in MSCs and NIM-1-induced cells. After differentiation with NIM-2 protocols the Cx43 expression was reduced compared to MSCs. Cx26, which was the only up-regulated connexin at mRNA level after differentiation, was barely detectable in MSCs and NIM-1-differentiated cells by Western blot, but upregulated after NIM-2 differentiation (Figure 3B). Immunocytochemistry staining of these connexins confirmed the Western blotting results showing cell membrane associated staining and gap junction plaque formation (Figure 3C).

**FIGURE 4:**
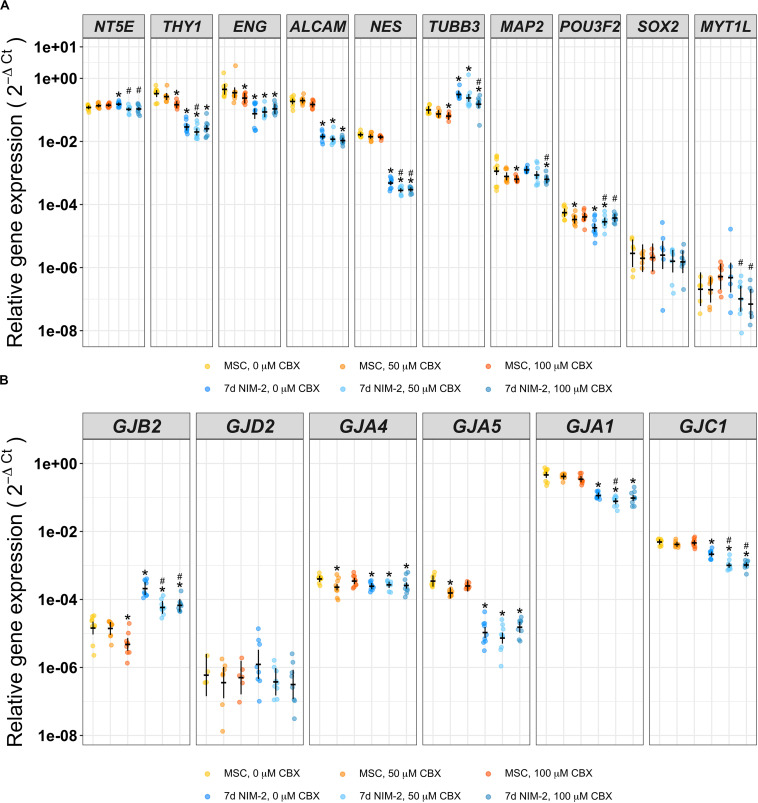
Differentiation of MSCs in presence of the gap junction inhibitor CBX. mRNA of MSCs supplemented with 0 μM, 50 μM or 100 μM CBX for 7 days and of 7 d NIM-2-treated MSCs supplemented with identical concentrations of CBX was analyzed by qRT-PCR. The original data points are plotted in colorful dots. The horizontal lines indicate model-based least-square means together with their 95%-confidence intervals (vertical lines). The * marks significant mean differences (α = 0.05) between MSC 0 μM and the other treatments while the # marks significant mean differences (α = 0.05) of 7 d NIM-2 in presence of 50 μM CBX and 7 d NIM-2 in presence of 100 μM CBX compared to 7 d NIM-2 in presence of 20 μM CBX. **(A)** qRT-PCR analysis of MSC and neuronal markers. **(B)** qRT-PCR analysis of *GJB2, GJA4*, *GJA5*, *GJA1*, *GJC1* and *GJD2*.

Different carbenoxolone (CBX) concentrations were added to NIM-2 during the 7 days differentiation to test whether gap junction blocking has an effect on the differentiation progress. In parallel, we added the identical concentrations of CBX to the MSC growth medium for 7 days as control. The gap junction inhibitor CBX did not influence the morphology of control MSCs nor the morphological changes induced by the 7 d NIM-2 protocol (data not shown). qRT-PCR experiments showed that *THY1*, *ENG*, *TUBB3*, *MAP2* and *POU3F2* were slightly reduced by addition of CBX in MSCs, while *NT5E*, *THY1*, *NES*, *TUBB3*, *MAP2*, *POU3F2* and *MYT1L* were altered in 7 d NIM-2-differentiated cells (Figure 4A). Concerning the connexin expression, *GJB2*, *GJA1* and *GJC1* were the connexins affected by CBX presence during 7 d NIM-2-induced transdifferentiation (Figure 4B).

## Discussion

In this study, gap junction mediated cell communication was studied during neuronal differentiation by analyzing the expression pattern of connexin isoforms and performing dye transfer experiments to study the functionality of the formed gap junction channels. To induce a neuronal transdifferentiation of human bone marrow-derived MSCs, we used and adjusted the small-molecule based NIM-1 and NIM-2 protocols by Bi et al. (2010) and Aguilera-Castrejon et al. (2017), who discussed the impact of the applied small molecules in detail, whereby the effects of single small molecules vary with concentration and composition (Qin et al., 2017). Analyzing the resulting morphological development, changes in the expression pattern of neuronal markers as well as action potential measurements of the developed cells allowed a characterization of the induced neuron-like cells.

All tested protocols were able to stop cell proliferation and induced a morphological development of MSCs towards neuron-like cell shapes with round cell bodies and long neurite-like processes. Similar morphological changes were observed when neuronal cells were induced either by transfection with neuronal lineage transcription factors (Vierbuchen et al., 2010; Pang et al., 2011) or by small molecules (Bi et al., 2010; Alexanian et al., 2013; Aguilera-Castrejon et al., 2017) and therefore represent a first indication for neuronal differentiation. Moreover, we found that the expression of typical MSC markers was reduced, which suggests a loss of mesenchymal stem cell properties and ongoing differentiation. The multi-lineage progenitor cell marker *NES* was down-regulated in NIM-2-differentiated cells while its level was not altered in NIM-1-differentiated cells. As the Nestin expression is down-regulated when neuronal stem cells stop proliferating and enter the neuronal differentiation (Zhang and Jiao, 2015) it could be possible that NIM-2-induced cells were further developed than the NIM-1-differentiated cells which more resembled neural progenitor cells. Underlining this assumption, the differentiation with the NIM-2 protocols up-regulated the immature neuronal marker Tuj1 at both mRNA (*TUBB3*) and protein level while the NIM-1 protocol did not change its expression. Although *MAP2* was increased at mRNA level after NIM-1 differentiation it could not be detected at protein level. Generally, the effects of the induction with NIM-2 were reinforced when the differentiation time was extended to 30 days. The maturation did not perceivably alter the marker expression pattern of the differentiated cells but stabilized the cells and enabled a stable whole-cell patch-clamp configuration to perform electrophysiological measurements and induce action potentials. These action potentials lasted longer than expected from neurons (Hodgkin and Huxley, 1952; Jiang et al., 2015) and were neither strongly overshooting nor repetitive. They more resembled action potentials measured in immature neurons (Ma et al., 2011; Connor et al., 2018), smooth muscle cells or cardiomyocytes (Voitychuk et al., 2012; Manchanda et al., 2019). However, a myogenic differentiation was considered unlikely as muscle specific markers were not upregulated at mRNA level (Supplementary Figure 1). Taken together, our data indicate an appreciable degree of neuronal differentiation as shown before by Bi et al. (2010) and Aguilera-Castrejon et al. (2017). The cells appear further developed than neuronal stem or progenitor cells although they do not show the characteristics of fully mature neurons. These induced cells therefore offered the possibility for further developmental studies of connexin expression and gap junction coupling in MSCs overcoming the mesenchymal cell fate.

Dye transfer experiments revealed that undifferentiated MSCs were well gap junction-coupled, which is consistent with the findings of other authors who showed extensive gap junction coupling in MSCs (Dorshkind et al., 1993; Valiunas et al., 2004). Although neurons are also coupled by gap junctions (Gutnick and Prince, 1981; Noctor et al., 2001) we saw a significant reduction of the LY dye coupling rate in NIM-2-differentiated cells suggesting a reduction in the number of gap junction channels what has already been observed in neurons (Eugenin et al., 2012). The gap junction coupling rate of the NIM-1-induced neural progenitor-like cells was not altered compared to the control MSCs. This finding is consistent with the conception that gap junction coupling is abundant and essential for neuronal stem and progenitor cells (Duval et al., 2002) as gap junction coupling is a crucial element for cell survival and the maintenance of the self-renewal state of stem cells (Cheng et al., 2004; Todorova et al., 2008).

Cx43, Cx40 and Cx45 play the major role in the gap junction coupling capabilities of MSCs (Bodi et al., 2004; Valiunas et al., 2004). We expected that the differentiation of MSCs into neuron-like cells would down-regulate mesenchymal connexins like Cx43, Cx40 and Cx37 while up-regulating neuronal connexins like Cx26, Cx45 and especially Cx36 (Rozental et al., 2000; Söhl et al., 2005; Eugenin et al., 2012; Su et al., 2017). Our analysis of connexin expression in the transdifferentiated neuron-like cells showed a significant down-regulation of the mesenchymal connexins Cx43, Cx40 and Cx37 and an upregulation of Cx26, thereby confirming our expectations. These changes in the connexin expression pattern correlated with a reduction of the gap junction coupling rate showing that the upregulation of Cx26 alone was not sufficient to maintain a high gap junction coupling rate. The presence of Cx26 *in vivo* is essential for a proper neuronal development, synapse formation in the neocortex and animal behavior (Su et al., 2017) and could be considered as another indicator for neuronal development of the MSCs. Cx36 is predominantly found in neurons. It has important roles in synapse activities and in the developing brain (Condorelli et al., 2000; Gulisano et al., 2000). Its presence would therefore be expected in fully developed neurons but was not found after transdifferentiation, indicating an incomplete differentiation. For Cx43 it has been shown that its extensive expression in human neural progenitor cells (Rozental et al., 2000) promoted growth factor dependent proliferation and repressed neural differentiation (Lemcke and Kuznetsov, 2013). To promote neuronal differentiation Cx43 has to be down-regulated (Rinaldi et al., 2014) and an uncoupling of the neural progenitor cells has to take place (Rozental et al., 2000). We could measure a significant decrease of Cx43 level although it was still stronger expressed than the other analyzed connexins after differentiation. Despite incomplete down-regulation of mesenchymal connexins, the reduction of gap junction coupling could indicate a differentiation progress towards a neuronal fate, since the suppression of gap junction coupling is described as prerequisite for exiting the cell cycle and inducing differentiation (Rozental et al., 2000; Lemcke and Kuznetsov, 2013; Lemcke et al., 2013; Rinaldi et al., 2014). As the unspecific gap junction inhibitor CBX, which is known to efficiently and rapidly close gap junction channels, did not accelerate the NIM-2-induced differentiation, it seems that the reduction of gap junction coupling might be necessary for complete neuronal development but was no promoting signal for the induction of differentiation.

## Conclusion

We could show an electrophysiological excitability of neuronal-like cells derived by a small molecule-based conversion of human MSCs into cells of neuronal lineage. MSC markers were down-regulated and the neuronal marker Tuj1 was up-regulated during differentiation while the cells developed a neuron-like morphology and, more importantly, physiological and functional properties of early neuronal cells. The connexin expression was modulated during differentiation while the gap junction coupling of the induced neuronal cells was almost entirely suppressed. This reduction of gap junction coupling is a prerequisite for neuronal differentiation but appears to be no driving mechanism itself. The here presented transdifferentiation approach towards neuron-like cells, however, revealed severe limitations both in phenotypic (gene and protein expressions) and in functional abilities of the cells. In the future, apart from small molecules, transcription factor-based reprogramming might be pursued as a more efficient and stable alternative.

## Data Availability Statement

All datasets generated for this study are included in the article/Supplementary Material. Additionally, all datasets and corresponding R-code are available under https://github.com/MaxMenssen/Dilger_et_al_2020.

## Ethics Statement

The studies involving MSCs isolated from bone marrow of a human donor were reviewed and approved by the Ethics committee of Hannover Medical School. The donor provided written informed consent to donate tissue.

## Author Contributions

ND and AN designed the research and wrote the manuscript. ND, A-LN, and KG performed and analyzed the experiments. AH isolated and provided the MSCs. A-LN, KG, AH, and MM contributed to the data interpretation and revised the manuscript. MM was responsible for the data processing, as well as the statistical analysis. The manuscript was proofread by all authors. All authors contributed to the article and approved the submitted version.

## Conflict of Interest

The authors declare that the research was conducted in the absence of any commercial or financial relationships that could be construed as a potential conflict of interest.
