# Supplementary material for: Gap Junction Dependent Cell Communication Is Modulated During Transdifferentiation of Mesenchymal Stem/Stromal Cells Towards Neuron-Like Cells
Source: Front Cell Dev Biol. 2020 Aug 31;8:869. doi: 10.3389/fcell.2020.00869 (PMC7487424; doi:10.3389/fcell.2020.00869)
Supplement: Supplementary file 1 [file Data_Sheet_1.PDF]

Supplementary Material

1 Supplementary Figures and Tables

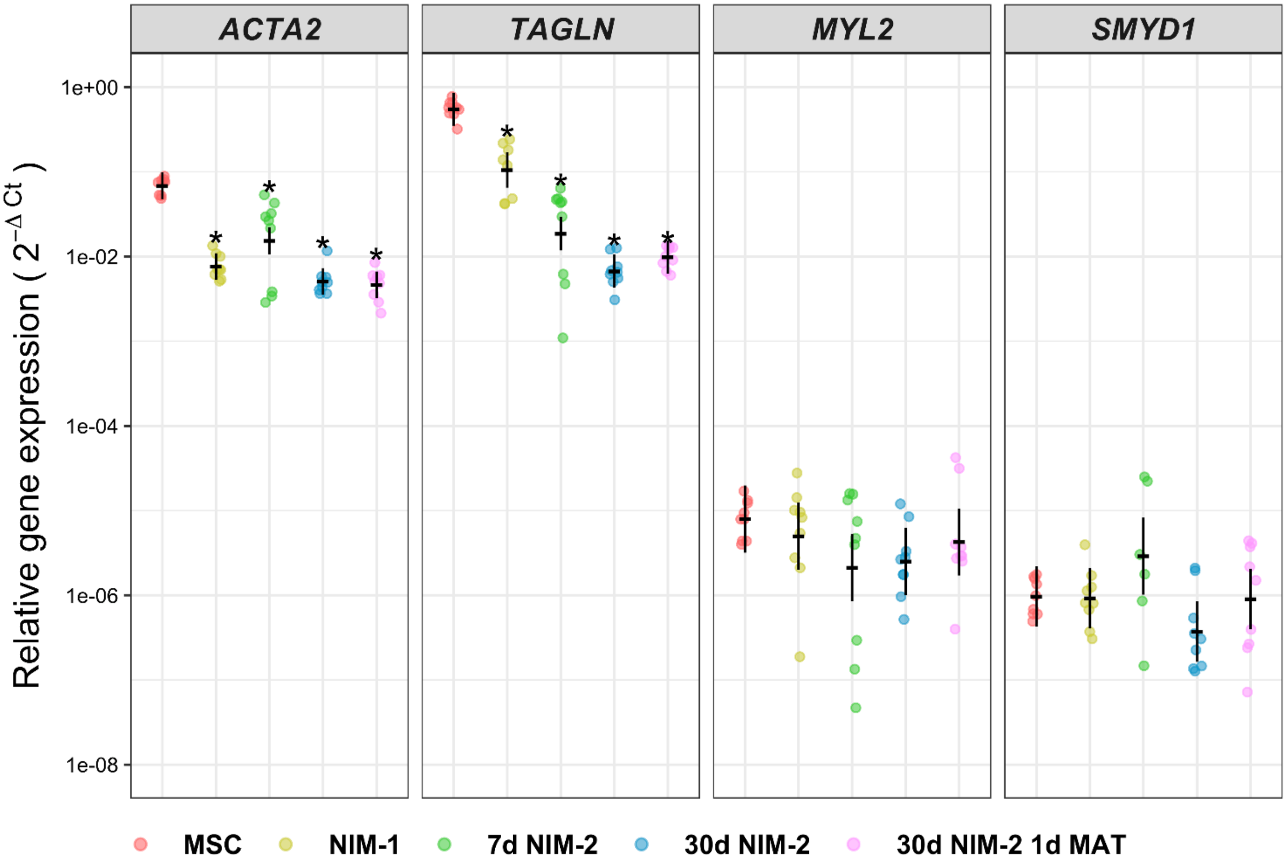

Supplemental Figure 1: Expression analysis of myogenic markers in untreated MSCs. The markers *ACTA2* and *TAGLN* were expressed in MSCs and were down-regulated after differentiation. *MYL2* and *SMYD1* showed a weak expression level in control MSCs and remained at a low level after differentiation or were down-regulated. All single data points are plotted in colorful dots. The horizontal lines indicate the linear model-based least-square mean values while the vertical lines represent the confidence intervals with $\alpha = 0.05$. Significant differences compared to MSCs are indicated as *.

Supplemental Table 1: Neuronal differentiation media.

NIM-1	NIM-2	MAT
DMEM/Ham's F12 (1:1)	50 % DMEM/Ham's F12 (1:1)	50 % DMEM/Ham's F12 (1:1)
100 U/mL penicillin	50 % Neurobasal™ medium	50 % Neurobasal™ medium
0.1 mg/mL streptomycin	100 U/mL penicillin	100 U/mL penicillin
1.6 mM valproic acid	0.1 mg/mL streptomycin	0.1 mg/mL streptomycin
1.6 % DMSO	1x N-2 supplement	0.5x N-2 supplement
160 µM butylhydroxyanisole	1x B-27™ supplement	1x B-27™ supplement
8 µM forskolin	1x GlutaMAX™	20 ng/mL human FGF-2 (100-18B)
0.8 µM hydrocortisone	1x non-essential amino acids	20 ng/mL BDNF (PeproTech, 450-02)
20 mM KCl	100 µM dbcAMP	20 ng/mL GDNF (PeproTech, 450-10)
4 µg/mL insulin	5 µg/mL heparin	20 ng/mL NT-3 (PeproTech, 450-03)
	1 µM I-BET	3 µM CHIR99021
	20 µM CHIR99021	10 µM forskolin
	1 µM Repsox	1 µM dorsomorphin
	50 µM forskolin	100 µM dbcAMP
	5 µM Y-27632	
	20 ng/mL human FGF-2 (PeproTech, 100-18B)	

Supplemental Table 2: Protein and gene equivalents.

Gene name	Protein name	Long name
RPS29	Rps29	40S ribosomal protein S29
GAPDH	GAPDH	Glyceraldehyde-3-phosphate dehydrogenase
NT5E	CD73	Ecto-5'-nucleotidase
THY1	CD90	Thy-1 cell membrane glycoprotein
ENG	CD105	Endoglin
ALCAM	CD166	Activated leukocyte cell adhesion molecule
NES	Nestin	Nestin
TUBB3	Tuj1	neuron-specific class III beta-tubulin
RBFOX3	NeuN	Neuronal nuclei antigen
MAP2	MAP-2	Microtubule-associated protein 2
POU3F2	Brn-2	Brain-specific homeobox/POU domain protein 2
SOX2	SOX-2	Transcription factor SOX-2
MYT1L	MyT1-L	myelin transcription factor 1-like protein
GJB2	Cx26	Connexin26
GJD2	Cx36	Connexin36
GJA4	Cx37	Connexin37
GJA5	Cx40	Connexin40
GJA1	Cx43	Connexin43
GJC1	Cx45	Connexin45
ACTA2	α-actin-2	Alpha-actin-2
TAGLN	Transgelin	Transgelin
MYL2	Myl2	myosin light chain 2
SMYD1	SmyD1	Histone-lysine N-methyltransferase SMYD1

Supplemental Table 3: Primer pairs used for gene expression analysis by qRT-PCR (annealing temperature 60 °C).

Target gene		Primer sequence 5'-3'	Amplicon size (bp)	Efficiency (%)
RPS29	Forward	CGAAAATTCGGCCAGGGTTC	109	94
	Reverse	TCGCGTACTGACGGAAACAC		
NT5E	Forward	GGAACCACGTATCCATGTGC	76	93
	Reverse	TGGATTCCATTGTTGCGTTCA		
THY1	Forward	GCGTCTGGAGGAGGCTG	117	93
	Reverse	GGGAGACCTGCAAGACTGTTA		
ENG	Forward	CCTGACCTGTCTGGTTGCAC	126	96
	Reverse	ACGCGTGTGCGAGTAGATG		
ALCAM	Forward	ACGATGAGGCAGACGAGATAAG	150	93
	Reverse	ATGCAGTCTTTGACTTCTTCATGT		
NES	Forward	TCAGCTTTCAGGACCCCAAG	130	91
	Reverse	GGTGTCTCAAGGGTAGCAGG		
TUBB3	Forward	AGCAAGAACAGCAGCTACTTCGT	102	96
	Reverse	GATGAAGGTGGAGGACATCTTGA		
RBFOX3	Forward	CTCCGCCTGAAGCGATGG	115	95
	Reverse	GAGTAGTCCTGCGTGGGGT		
MAP2	Forward	AGTTCCAGCAGCGTGATG	95	98
	Reverse	CATTCTCTCTTCAGCCTTCTC		
POU3F2	Forward	CTGGTGCAGGGCGACTAC	73	92
	Reverse	CGGTGATCCACTGGTGAG		
SOX2	Forward	CAAGATGCACAACTCGGAGA	147	98
	Reverse	CGGGGCCGGTATTTATAATC		
MYT1L	Forward	ATCAAGCCATGGAAACTTGG	126	94
	Reverse	TCCACCTCTGATAAGATCCT		
GJB2	Forward	CTCCCGACGCAGAGCAAA	114	90
	Reverse	ACGGTGAGCCAGATCTTTCC		
GJD2	Forward	AAGGCATCTCCCGCTTCTACA	71	98
	Reverse	GCCAACCAGGAACCCAATTT		
GJA4	Forward	CAGGCAGGCGACGGAG	127	109
	Reverse	TGCGGAAGATGAAGAGCACC		
GJA5	Forward	GCAGCCAGAGTGTGAAGAAG	150	110
	Reverse	CGGTCGAGTGCTTGTGTACT		
GJA1	Forward	TTTAAGCAAAAGAGTGGTGCCC	98	103
	Reverse	CTTCCCTCCAGCAGTTGAGT		
GJC1	Forward	CTGGACAACAGGGCATACCA	144	93
	Reverse	ACAGCTGTAAGGACGATCCG		
ACTA2	Forward	AAAAGACAGCTACGTGGGTGA	76	93
	Reverse	GCCATGTTCTATCGGGTACTTC		
TAGLN	Forward	CTATGGCATGAGCCGCGAAG	97	95
	Reverse	GCCACACTGCACTATGATCCA		
MYL2	Forward	TCTTTGCAGAGTGGTCCCTG	140	104
	Reverse	GCCACGAAGTACCCATAGCC		
SMYD1	Forward	CAGAACCACGTGGAGCACTT	130	107
	Reverse	CTCCGAAGATGTGCGAGATGT		
